# Supplementary material for: Origins and impact of extrachromosomal DNA
Source: Nature. 2024 Nov 6;635(8037):193–200. doi: 10.1038/s41586-024-08107-3 (PMC11540846; doi:10.1038/s41586-024-08107-3)
Supplement: Supplementary file 1 — A description of ecDNA calls, a summary of Supplementary Tables 1–11 and additional references. [file 41586_2024_8107_MOESM1_ESM.pdf]

nature portfolio

https://doi.org/10.1038/s41586-024-08107-3

Supplementary information

Origins and impact of extrachromosomal DNA

In the format provided by the authors and unedited

Supplementary Information Guide

Table of Contents

ecDNA calls.......... 1
AmpliconArchitect 1
CNVkit.......... 1
Supplementary Table Information 2
Supplementary Table 1 2
Supplementary Table 2 2
Supplementary Table 3 2
Supplementary Table 4 2
Supplementary Table 5 2
Supplementary Table 6 4
Supplementary Table 7 3
Supplementary Table 8 3
Supplementary Table 9 3
Supplementary Table 10 2

ecDNA calls

AmpliconArchitect

A singularity container (accessed in the Genomics England shared environment under /re_gecip/shared_allGeCIPs/pancancer_ecdna/singularity_containers) containing Amplicon Architectv1.2 was run using the following command and flags:

singularity exec --bind /genomes/by_date re_gecip/shared_allGeCIPs/pancancer_ecdna/singularity_containers/prepare_aa_may2022.sif python /home/programs/PrepareAA-master/PrepareAA.py --cnvkit_dir /home/programs/cnvkit.py -o $OUTDIR -s $SAMPLE -t 4 --ref GRCh38 --sorted_bam $BAMFILE --normal_bam $NORMAL --downsample 10 --run_AA --run_AC

CNVkit

Sample copy number was rescaled posthoc using cnvkit version 0.98 using the command cnvkit.py call -m none $SAMPLE.cns --purity $PURITY -y -o $SAMPLE.rescaled.cns

Population Characteristics

For the Cox proportional hazards model, patients were grouped according to sex (biological attribute; male = 3262, female = 5615) and age groups (0-44 years, n = 481; 45-59 years, n = 2215; 60 - 69 years, n = 2648; 70-79 years, n = 2581; 80+ years, n = 951). Only samples with complete staging information were included in the model, and tumour types with a cohort size > 100.

Supplementary Table Information

A summary of the supplementary tables are provided below.

Supplementary Table 1

Count of ecDNA in GEL cohort. The tissue type and cancer types are both supplied. 95 % confidence intervals of the proportions are calculated using the R function prop.ci().

Supplementary Table 2

The number of ecDNA calls that feature a specific oncogene by cancer type. The oncogene table was downloaded from https://cancer.sanger.ac.uk/census (accessed 10/14/2022) and was filtered for those genes where the 'Role in Cancer' contained the keyword 'oncogene'.

Supplementary Table 3

ecDNA are presented by estimated copies per cell and estimated size in kilobase pairs (kbp) with interquartile ranges given for both.

Supplementary Table 4

Estimated oncogene copies per cell and estimated ecDNA size in kilobase pairs (kbp) with interquartile ranges given.

Supplementary Table 5

Gene focal amplification dN/dS estimates by amplification type. dN/dS point mutation estimates were calculated using the dNdScv package, with estimates for missense and truncating mutations provided.

Supplementary Table 6

Immunomodulatory gene list and ecDNA frequency. Genes were classified as Immunomodulatory if they met the following gene ontology GO terms: 0006968 (cellular defense response), 0002228 (natural killer cell mediated immunity), 0042267 (natural killer cell mediated cytotoxicity), 0001906 (cell killing), 0001909 (leukocyte mediated cytotoxicity), 0002698 (negative regulation of immune effector process), 0001910 (regulation of leukocyte mediated cytotoxicity), 0031341

(regulation of cell killing), 0002367 (cytokine production involved in immune response), 0002695 (negative regulation of leukocyte activation), 0050866 (negative regulation of cell activation), 0051250 (negative regulation of lymphocyte activation), 0050777 (negative regulation of immune response), and an ecDNA was considered immunomodulatory only if it did not also contain an oncogene within the predicted locus. Immunomodulatory genes are also categorised according to whether or not the ecDNA from which they reside contain both immunomodulatory genes and oncogenes.

Supplementary Table 7

The odds ratio of ecDNA presence by tumour suppressor gene. The tumour suppressor gene table was downloaded from https://cancer.sanger.ac.uk/census (accessed 10/14/2022) and was filtered for those genes where the 'Role in Cancer' contained the keyword 'TSG'. In this analysis, tumour suppressor mutations that were identified in each sample were characterised according to its calculated variant consequences (https://useast.ensembl.org/info/genome/variation/prediction/predicted_data.html). Those mutations categorised as high impact (transcript ablation, splice acceptor variant, splice donor variant, stop gained, frameshift variant, stop lost, start lost, transcript amplification, feature elongation and feature truncation) were included in the analysis. A multivariable logistic regression model was performed on a matrix that detailed the presence or absence of ecDNA and the presence of absence of a specific tumour suppressor gene mutation per sample.
For the overall cohort analysis, a tumour suppressor gene had to be mutated in at least 20 samples in two separate tumour types with a univariate q-value > 0.01 to be included. Tumour type analysis was performed with cohorts with over 100 samples. A tumour suppressor mutation had to occur in at least 20 samples with a univariate q-value > 0.05 to be included.

Supplementary Table 8

Global dN/dS estimates by cancer gene and amplification type. The cancer gene list was downloaded from https://cancer.sanger.ac.uk/census. dN/dS point mutation estimates were calculated using the dNdScv package per gene. The table gives the frequency of synonymous, missense and non-synonymous mutations per gene with missense, nonsense, indel and splice site dN/dS estimates and q values for missense, truncated and global estimates.

Supplementary Table 9

The odds ratio of ecDNA presence by the weighted genome instability index (wGii), structural variant (SV) burden and whole genome duplication (WGD) according to tumour type. Tumour type analysis was performed with cohorts with over 100 samples. wGii was calculated as the proportion of the genome with aberrant copy number relative to the median ploidy, weighted on a per chromosome basis. For this, the relative ploidy, and proportion of losses and gains were calculated from the .cns files from CNVkit. A sample was classified has having WGD if the sample ploidy was over 2.7 (https://cancer.sanger.ac.uk/cosmic/help/cnv/overview). For ploidy estimates, the CakeTin pipeline from Everall et al.[2] was utilized, available for 9,141

samples. Structural variant burden was calculated by dividing the number of SVs by 2,800, per sample. SV calls were obtained from the Genomics England research environment (https://re-docs.genomicsengland.co.uk/genomic_data/). The workflow generated SVs using Manta (version 0.28.0). Samples were categorised as having wGii high/low, SV burden high/low and WGD present/absent. A logistic regression was then performed to using ecDNA presence, tumour purity, wGii, SV burden and WGD as covariates.

Supplementary Table 10

The proportion of mutations attributed to a specific SBS process was estimated using a maximum likelihood function. This involves creating a mutational catalog that categorizes all mutations based on 96 trinucleotide context channels. Using the sample-level single base substitution exposures and the SBS reference signatures provided in Degasperi et al.[1], each trinucleotide channel context is assigned a likelihood value by multiplying the sample exposure weight by the reference signature weight, allowing for estimation of the most likely mutational process for each mutation. For each sample, the proportion of mutations attributed to a SBS process across the whole genome (globally) was compared with the proportion of mutations attributed to a SBS process within an ecDNA or chromosomal amplification *and* with adjacent flanks in the 3' and 5' direction totalling an identical size to the ecDNA for comparison. Wilcoxon p-values test were calculated from those comparisons.

References

1. Degasperi, A. *et al.* Substitution mutational signatures in whole-genome–sequenced cancers in the UK population. *Science* **376**, abl9283 (2022) doi:10.1126/science.abl9283.

2. Everall, A. *et al. Comprehensive Repertoire of the Chromosomal Alteration and Mutational Signatures across 16 Cancer Types from 10,983 Cancer Patients*. http://medrxiv.org/lookup/doi/10.1101/2023.06.07.23290970 (2023) doi:10.1101/2023.06.07.23290970.
