# Supplementary material for: Origins and impact of extrachromosomal DNA
Source: Nature. 2024 Nov 6;635(8037):193–200. doi: 10.1038/s41586-024-08107-3 (PMC11540846; doi:10.1038/s41586-024-08107-3)
Supplement: Supplementary file 2 — Reporting Summary [file 41586_2024_8107_MOESM2_ESM.pdf]

nature portfolio

Corresponding author(s): Charles Swanton

Last updated by author(s): Aug 2, 2024

Reporting Summary

Nature Portfolio wishes to improve the reproducibility of the work that we publish. This form provides structure for consistency and transparency in reporting. For further information on Nature Portfolio policies, see our Editorial Policies and the Editorial Policy Checklist.

Statistics

For all statistical analyses, confirm that the following items are present in the figure legend, table legend, main text, or Methods section.

n/a	Confirmed	
☐	☒	The exact sample size (n) for each experimental group/condition, given as a discrete number and unit of measurement
☐	☒	A statement on whether measurements were taken from distinct samples or whether the same sample was measured repeatedly
☐	☒	The statistical test(s) used AND whether they are one- or two-sided<br>*Only common tests should be described solely by name; describe more complex techniques in the Methods section.*
☐	☒	A description of all covariates tested
☐	☒	A description of any assumptions or corrections, such as tests of normality and adjustment for multiple comparisons
☐	☒	A full description of the statistical parameters including central tendency (e.g. means) or other basic estimates (e.g. regression coefficient) AND variation (e.g. standard deviation) or associated estimates of uncertainty (e.g. confidence intervals)
☐	☒	For null hypothesis testing, the test statistic (e.g. F, t, r) with confidence intervals, effect sizes, degrees of freedom and P value noted<br>*Give P values as exact values whenever suitable.*
☒	☐	For Bayesian analysis, information on the choice of priors and Markov chain Monte Carlo settings
☐	☒	For hierarchical and complex designs, identification of the appropriate level for tests and full reporting of outcomes
☐	☒	Estimates of effect sizes (e.g. Cohen's d, Pearson's r), indicating how they were calculated

Our web collection on statistics for biologists contains articles on many of the points above.

Software and code

Policy information about availability of computer code

Data collection

No software was used

Data analysis

Focal DNA copy number alterations were identified using CNVKit v0.98. Amplicon Architect v1.2 was used to construct cyclic paths from identified focal amplifications, and Amplicon Classifier version 0.4.12 was used to determine whether these paths were likely to be ecDNA. Amplicon Architect is a tool that identifies the structure of focal amplifications by using seed intervals that define regions that are focally amplified and extend beyond them to look for copy number changes or discordant edges. For this analysis, focal amplifications were defined as regions of over 50Kb, with a copy number greater than four and twice the ploidy estimation of the chromosome arm.

R packages used in version 4.0.2:
survival (v3.2.11)
survminer (v0.4.9)
fst (version 0.9.4)
tidyverse (version 1.3.0)
ggplot2 (version 3.3.2)
dplyr (version 1.0.2)
tidyr (version 1.1.2)
gridExtra (version 2.3)
cowplot (version 1.1.0)
ggpubr (version 0.4.0)
reshape2 (version 1.4.4)
tibble (version 3.0.4)

RColorBrewer (version 1.1-2)
plyr (version 1.8.6)
dndscv (version 0.0.1.0)
deconstructSigs (version 1.9.0)
ggrepel (version 0.8.2)
GenomicRanges (version 1.38.0)
stringr (version 1.4.0)
data.table (version 1.13.2)
magrittr (version 2.0.1)
ComplexHeatmap (version 2.4.5)

For manuscripts utilizing custom algorithms or software that are central to the research but not yet described in published literature, software must be made available to editors and reviewers. We strongly encourage code deposition in a community repository (e.g. GitHub). See the Nature Portfolio guidelines for submitting code & software for further information.

Data

Policy information about availability of data

All manuscripts must include a data availability statement. This statement should provide the following information, where applicable:

- Accession codes, unique identifiers, or web links for publicly available datasets
- A description of any restrictions on data availability
- For clinical datasets or third party data, please ensure that the statement adheres to our policy

All data is available following application to access to the Genomics England Research Environment.

Research involving human participants, their data, or biological material

Policy information about studies with human participants or human data. See also policy information about sex, gender (identity/presentation), and sexual orientation and race, ethnicity and racism.

Reporting on sex and gender	Sex (biological attribute) was used as an explanatory variable in the Cox proportional hazards model for the association with ecDNA presence and survival
Reporting on race, ethnicity, or other socially relevant groupings	We did not report on race, ethnicity or other socially relevant groupings
Population characteristics	For the Cox proportional hazards model, patients were grouped according to sex (biological attribute; male = 3262, female = 5615) and age groups (0-44 years, n = 481; 45-59 years, n = 2215; 60 - 69 years, n = 2648; 70-79 years, n = 2581; 80+ years, n = 951)
Recruitment	Patients were recruited to eleven Genomic Medicine Centres across the United Kingdom;<br><br>- East of England NHS Genomic Medicine Centre (Led by Cambridge University Hospitals NHS Foundation Trust)<br>- Greater Manchester NHS Genomic Medicine Centre (Led by Central Manchester University Hospitals NHS Foundation Trust)<br>- West Midlands NHS Genomic Medicine Centre (Led by University Hospitals Birmingham NHS Foundation Trust)<br>- North East & North Cumbria NHS Genomic Medicine Centre (Led by Newcastle upon Tyne Hospitals NHS Foundation Trust)<br>- North Thames NHS Genomic Medicine Centre (Led by Great Ormond Street Hospital NHS Foundation Trust)<br>- North West Coast NHS Genomic Medicine Centre (Led by Liverpool Women’s NHS Foundation Trust)<br>- Oxford NHS Genomic Medicine Centre (Led by Oxford University Hospitals NHS Trust (OUH))<br>- South London NHS Genomic Medicine Centre (Led by Guy’s and St Thomas’ NHS Foundation Trust)<br>- South West NHS Genomic Medicine Centre (Led by Royal Devon & Exeter NHS Foundation Trust)<br>- Wessex NHS Genomic Medicine Centre (Led by University Hospital Southampton NHS Foundation Trust)<br>- West London NHS Genomic Medicine Centre (Led by Imperial College Healthcare NHS Trust)<br><br>Data release version 11 was used for the analysis and was launched in 17/12/2020, from which 15,609 participants are included.
Ethics oversight	Genomics England has approval from the HRA Committee East of England – Cambridge South (REC Ref 14/EE/1112).

Note that full information on the approval of the study protocol must also be provided in the manuscript.

Field-specific reporting

Please select the one below that is the best fit for your research. If you are not sure, read the appropriate sections before making your selection.

☒ Life sciences ☐ Behavioural & social sciences ☐ Ecological, evolutionary & environmental sciences

For a reference copy of the document with all sections, see nature.com/documents/nr-reporting-summary-flat.pdf

Life sciences study design

All studies must disclose on these points even when the disclosure is negative.

Sample size	We utilised the Genomics England version 7 cohort, comprising 16,355 whole genome sequenced samples. We included the following tumour types: breast, lung, stomach, neuroendocrine, skin, oropharyngeal, colorectal, kidney, prostate, hepato-pancreatobiliary, bladder, bone and soft tissue, ovary, endometrium, central nervous system (CNS), lymphoid and myeloid.
Data exclusions	For quality control, we excluded samples with an estimated purity of < 10%. Paediatric and germ cell tumours were also excluded. For mutational signature analysis the tumour purity cut off was 20%.
Replication	All available data was analysed.
Randomization	Randomisation is not relevant as this is an observational study.
Blinding	Blinding is not relevant as this is an observational study

Reporting for specific materials, systems and methods

We require information from authors about some types of materials, experimental systems and methods used in many studies. Here, indicate whether each material, system or method listed is relevant to your study. If you are not sure if a list item applies to your research, read the appropriate section before selecting a response.

Materials & experimental systems

n/a	Involved in the study	
☒	☐	Antibodies
☒	☐	Eukaryotic cell lines
☒	☐	Palaeontology and archaeology
☒	☐	Animals and other organisms
☒	☐	Clinical data
☒	☐	Dual use research of concern
☒	☐	Plants

Methods

n/a	Involved in the study	
☒	☐	ChIP-seq
☒	☐	Flow cytometry
☒	☐	MRI-based neuroimaging

Plants

Seed stocks	NA
Novel plant genotypes	NA
Authentication	NA
